# The Low-pH Stability Discovered in Neuraminidase of 1918 Pandemic Influenza A Virus Enhances Virus Replication

**DOI:** 10.1371/journal.pone.0015556

**Published:** 2010-12-09

**Authors:** Tadanobu Takahashi, Yuuki Kurebayashi, Kumiko Ikeya, Takashi Mizuno, Keijo Fukushima, Hiroko Kawamoto, Yoshihiro Kawaoka, Yasuo Suzuki, Takashi Suzuki

**Affiliations:** 1 Department of Biochemistry, University of Shizuoka, School of Pharmaceutical Sciences and Global COE Program for Innovation in Human Health Sciences, Shizuoka, Japan; 2 CREST, Japan Science and Technology Agency, Saitama, Japan; 3 Division of Virology, Department of Microbiology and Immunology, Institute of Medical Science, University of Tokyo, Tokyo, Japan; 4 Department of Pathobiological Sciences, University of Wisconsin, Madison, Wisconsin, United States of America; 5 Department of Biomedical Sciences, College of Life and Health Sciences, Chubu University, Kasugai-shi, Japan; University of Cambridge, United Kingdom

## Abstract

The “Spanish” pandemic influenza A virus, which killed more than 20 million worldwide in 1918-19, is one of the serious pathogens in recorded history. Characterization of the 1918 pandemic virus reconstructed by reverse genetics showed that PB1, hemagglutinin (HA), and neuraminidase (NA) genes contributed to the viral replication and virulence of the 1918 pandemic influenza virus. However, the function of the NA gene has remained unknown. Here we show that the avian-like low-pH stability of sialidase activity discovered in the 1918 pandemic virus NA contributes to the viral replication efficiency. We found that deletion of Thr at position 435 or deletion of Gly at position 455 in the 1918 pandemic virus NA was related to the low-pH stability of the sialidase activity in the 1918 pandemic virus NA by comparison with the sequences of other human N1 NAs and sialidase activity of chimeric constructs. Both amino acids were located in or near the amino acid resides that were important for stabilization of the native tetramer structure in a low-pH condition like the N2 NAs of pandemic viruses that emerged in 1957 and 1968. Two reverse-genetic viruses were generated from a genetic background of A/WSN/33 (H1N1) that included low-pH-unstable N1 NA from A/USSR/92/77 (H1N1) and its counterpart N1 NA in which sialidase activity was converted to a low-pH-stable property by a deletion and substitutions of two amino acid residues at position 435 and 455 related to the low-pH stability of the sialidase activity in 1918 NA. The mutant virus that included “Spanish Flu”-like low-pH-stable NA showed remarkable replication in comparison with the mutant virus that included low-pH-unstable N1 NA. Our results suggest that the avian-like low-pH stability of sialidase activity in the 1918 pandemic virus NA contributes to the viral replication efficiency.

## Introduction

The “Spanish” pandemic influenza A virus, which killed more than 20 million worldwide in 1918-19, is one of the serious pathogens in recorded history. Reverse genetics viruses with the 1918 virus genes have demonstrated that PB1 [1], [2], hemagglutinin (HA) [3], and neuraminidase (NA) genes [1], [4], [5] play critical roles in the high virulence and replication of the virus. Phylogenetic analysis of the gene sequences of the 1918 virus suggested that the virus originated from an avian source [6]. From analysis of N1 NA gene sequences, the 1918 virus NA gene has been suggested to share many characteristics with both mammalian and avian viruses. From analysis of N1 NA protein sequences, the 1918 virus NA gene was placed within and near the root of the avian clade [7]. However, there has been no study in which the origin of the 1918 virus NA gene was estimated by function or nature of the NA protein.

Influenza virus NA is important for not only the release of newly formed virions from infected cells but also the initiation of the viral infection [8]–[10]. We have reported that sialidase activity of most of human and swine epidemic viruses irreversibly disappeared in an acidic condition (pH 4.0–5.0). In comparison with them, all of the pandemic N2 NA viruses (“Asian Flu” in 1957 and “Hong Kong Flu” in 1968) and all types of duck viruses tested maintained low-pH stability of sialidase activity in the NAs [11], [12]. We have identified the N2 NA amino acids responsible for the low-pH stability of the A/Hong Kong/1/68 (H3N2) virus [13]. Four reverse-genetic viruses were generated from a genetic background of A/WSN/33 (H1N1) that included parental N2 NAs of 1968 pandemic (H3N2) and epidemic (H2N2) strains or their counterpart N2 NAs in which low-pH stability of the sialidase activity was changed by substitution of one or two amino acid residues. The transfectant viruses bearing low-pH-stable sialidase activity of N2 NA showed obvious replication efficiency both *in vitro* and *in vivo* in comparison with the viruses bearing low-pH-unstable sialidase activity and that the viral sialidase activity in late endosome/lysosome traffic enhanced influenza A virus replication [10].

In the present study, we made a comparison of the low-pH stability of sialidase activity between 1918 pandemic and epidemic virus N1 NAs by measuring sialidase activities of cells genetically expressing NAs. We found that the 1918 NAs had low-pH stability like that of pandemic virus N2NAs and avian virus NAs. The 1918 NAs are thought to have inherited the avian virus-like property at the protein level, although the sequence of NA genes had both characteristics of avian and mammalian viruses. Experimental data for chimeric N1 NAs showed that a deletion and substitutions of two amino acid residues in the 1918 pandemic virus NA were associated with the low-pH stability of sialidase activity of the NA. Additionally, reverse genetics viruses including the low-pH-stable or low-pH-unstable N1NA showed that the low-pH stability of N1NA enhanced virus replication. This is the first study showing that the low-pH stability of sialidase activity detected in the 1918 NA correlates with enhanced virus replication. The low-pH stability of the 1918 NA confirmed that the 1918 NA is consistent with avian virus in origin. Our studies suggest that the low-pH stability of NA contributes to the pandemic potential of human influenza A viruses.

## Results and Discussion

### Measurement of the low-pH stabilities of N1NA sialidase activities

**Figure 1:**
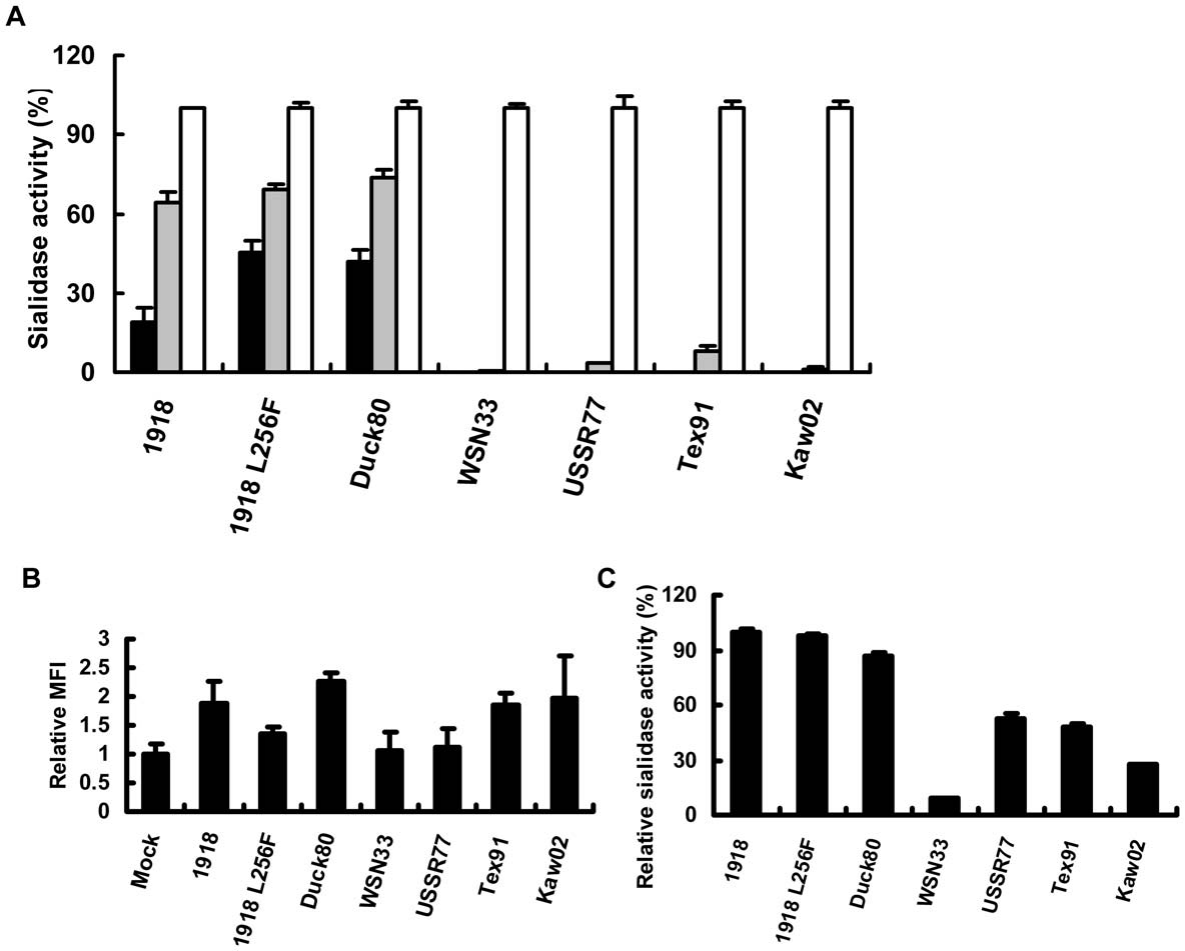
Low-pH stability of sialidase activity of the 1918 virus NA. A, Low-pH stabilities of N1 NA sialidase activities. N1 NA-expressing cells transfected with the NA genes of the 1918 virus (1918 and 1918 L256F), human H1N1 viruses (WSN33, USSR77, Tex91 and Kaw02) or duck H4N1 virus (Duck80) were incubated at pH 4.0 (closed column), 5.0 (hatched column) and 6.0 (open column) and sialidase activities were measured. Sialidase activities were expressed as a percentage of each activity at pH 6.0. B, Quantitation of NA expression on the cell surface. After fixation of cells with paraformaldehyde, NA expression on the cell surface was detected by rabbit anti-N1 NA polyclonal antibody and analyzed with a flowcytometer. Mean fluorescent intensity (MFI) was expressed as that relative to mock. C, Sialidase activity at NA expression levels of (A) under pH 6.0. Sialidase activities were expressed as a percentage of that of the 1918 virus NA.

To evaluate the low-pH stability of the 1918 NAs, we measured sialidase activities of cells genetically expressing each N1 NA from 1918 pandemic viruses, A/New York/1/18 or A/South Carolina/1/18 (1918NA) and A/Brevig Mission/1/18 (1918 L256FNA, GenBank accession number AF25036) [7], and each N1 NA from epidemic virus strains, A/WSN/33 (WSN33NA, GenBank accession number L25817), A/USSR/92/77 (USSR77NA, GenBank accession number CY009286), A/Texas/91 (Tex91NA, GenBank accession number CY009318) and A/Kawasaki/176/02 (Kaw02NA), and N1 NA from a duck virus strain, A/duck/849/3/80 (Duck80NA), after preincubation for 10 min under acidic conditions (pH 4.0 and 5.0). The 1918 virus NA gene had been sequenced from A/South Carolina/1/18, A/New York/1/18 and A/Brevig Mission/1/18, respectively [7]. One nucleotide (at position 788) are known to be heterogeneous within A/Brevig Mission/1/18 NA with two-thirds of clones having a C (phenylalanine at amino acid 256) and the rest an A (leucine at 256) at this site. We therefore used two types of 1918 virus NA genes (1918NA and 1918 L256FNA). 1918NA and 1918 L256FNA, but not WSN33NA, USSR77NA, Tex91NA and Kaw02NA, maintained sialidase activity under acidic conditions of pH 4.0 and 5.0 as did Duck80NA, which is representative of low-pH-stable NAs (Figure 1A). We measured NA expression on the cell surface by using a flow cytometer. Mean fluorescent intensities (MFI) of NA-expressing cells were 1 to 2-times higher than that of mock-treated cells (Figure 1B). Sialidase activities of NA-expressing cells were compared at the optimal condition (pH 6.0) as shown in Figure 1C. Although the amount of the low-pH stable 1918NA on the cell surface was similar to those of the low-pH unstable Tex91NA and Kaw02NA, sialidase activity of the 1918NA at pH 6.0 was about two-times higher than those of Tex91NA and Kaw02NA (Figure 1B–C). Absolute sialidase activity of 1918NA as well as Duck80NA also showed an avian virus-like property [14]. Calcium ions are known to be important for the enzyme activity of influenza virus NAs [15]. It has been reported that influenza viruses reach the early endosome after approximately 10 min of infection and late endosome with a low pH after 40–60 min [16]. Calcium uptake via endocytosis is known to rapidly release from acidifying endosomes. Ca^2+^ concentration in the endosomes of fibroblast cells after incubation for 10 minutes in a solution containing 2 mM CaCl_2_ was 3.9±1.2 µM [17]. We therefore measured the degree of low-pH stabilities of the NA-expressing cells with 10 mM acetate buffer (pH 4.0, 5.0, and 6.0) containing 5 µM CaCl_2_. We confirmed that the 1918 N1 NAs but not USSR77NA had avian-like low-pH stability under the condition (Figure S1).

### Degree of the low-pH stability of the 1918 NA

**Figure 2:**
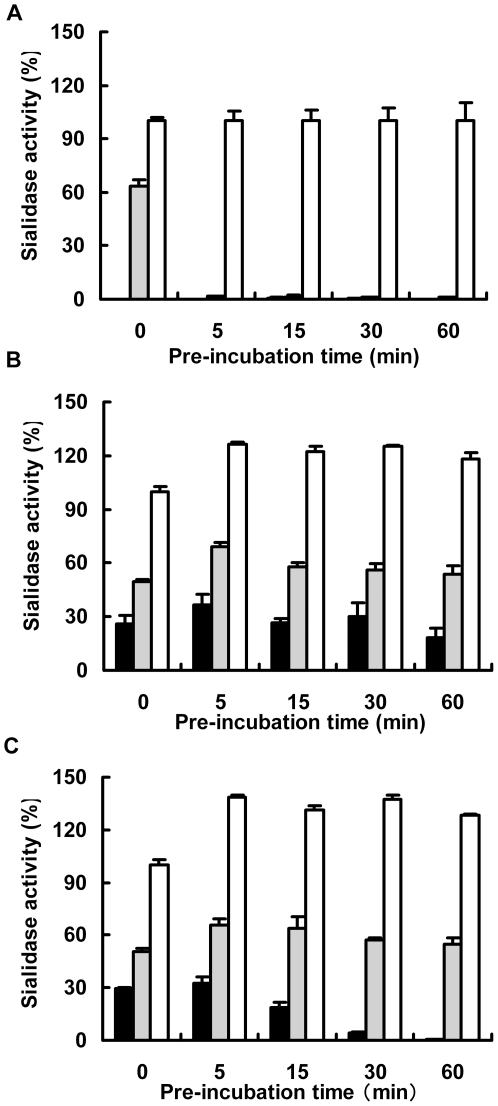
Time-dependency on the low-pH stability of sialidase activity of the 1918 virus NA. 293T cells genetically expressing each N1 NA from USSR77 (A), Duck80 (B) and 1918 (C) viruses were incubated for the indicated times at pH 4.0 (closed column), 5.0 (hatched column) and 6.0 (open column). Then sialidase activities were measured at each pH and expressed as a percentage of each activity at pre-incubation time of 0 min at pH 6.0.

We measured the degree of low-pH stability of the 1918 NA. NA-expressing cells were preincubated at pH 4.0, 5.0 and 6.0 for 0–60 min before addition of an enzymatic substrate. The cut-off values for assessing loss of the sialidase activity at pH 4.0 and 5.0 were determined by the activities of WSN33NA, USSR77NA, Tex91NA and Kaw02NA in Figure 1A. The cut- off values was 0.26 at pH 4.0 and 13.5 at pH 5.0, respectively. Sialidase activity of the low-pH unstable USSR77NA irreversibly disappeared at 0 min at pH 4.0 and 5 min at pH 5.0 (Figure 2A), but sialidase activity was maintained after preincubation for over 60 min at pH 4.0 and 5.0 in the low-pH stable Duck80NA (Figure 2B). Sialidase activity was maintained until preincubation for 30 min at pH 4.0 and for over 60 min at pH 5.0 in the NA of the 1918 virus (Figure 2C). The median values of the sialidase activity at pH 4.0 were compared between USSR77NA, Duck80NA and 1918NA. The median values of USSR77NA, Duck80NA and 1918NA were 0.19, 26.6 and 18.8, respectively. Taken together, these results indicate that the NA of the 1918 virus strains was originated from an avian source. Like human pandemic N2 viruses, the low-pH stability of the NA was thought to contribute to the pandemic potential for the 1918 virus. However, the low-pH stability of 1918NA was slightly weaker than that of Duck80NA, suggesting that the 1918 virus was adapting from avians to humans during the pandemic period in 1918–1919.

### Identification of amino acid residues responsible for the low-pH stability of the 1918 NA

**Figure 3:**
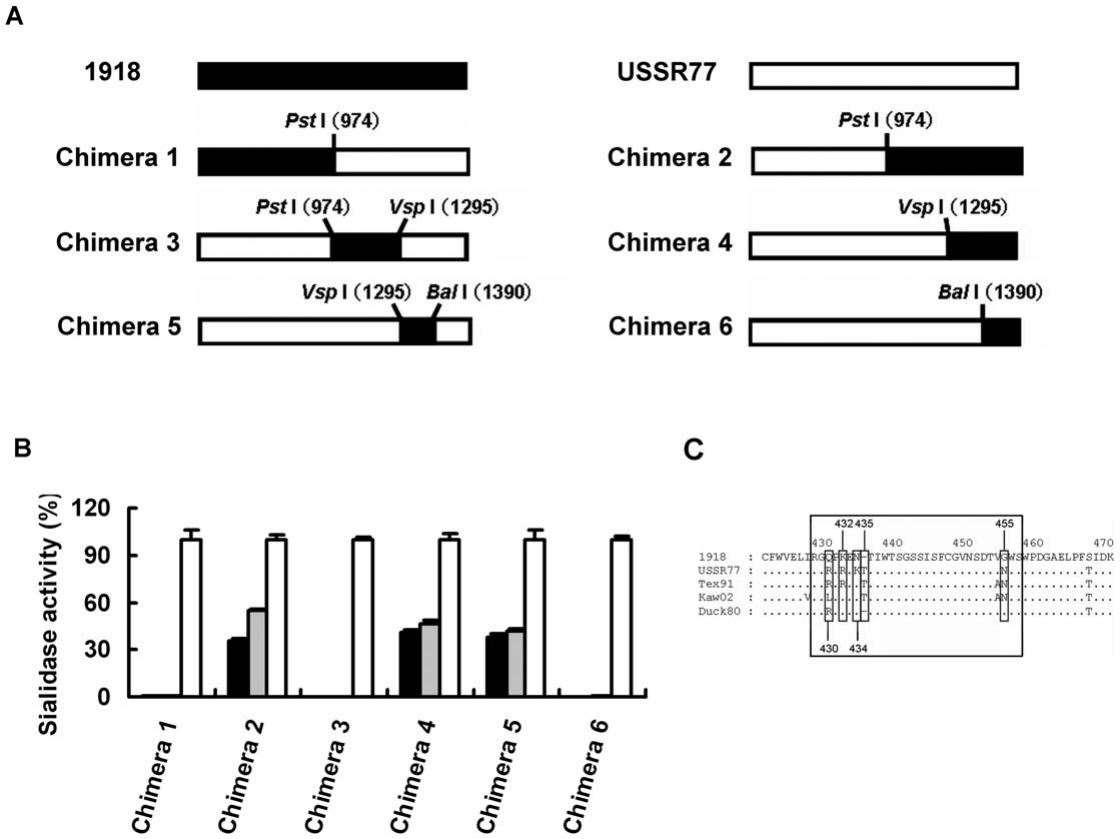
Low-pH stabilities of chimeric NAs between the 1918 NA and the USSR77 NA. A, Scheme of chimera NAs between the 1918 virus (1918) and USSR77. B, Low-pH stabilities of sialidase activities of chimera NAs. Sialidase activities were expressed as a percentage of each activity at pH 6.0. C, Comparison of NA amino acid sequences at positions 429 and 457 (USSR77 N1 numbering) among the viruses tested. c, Comparison of NA amino acid sequences of tested viruses at positions between 429 and 457 (USSR77 N1 numbering).

**Figure 4:**
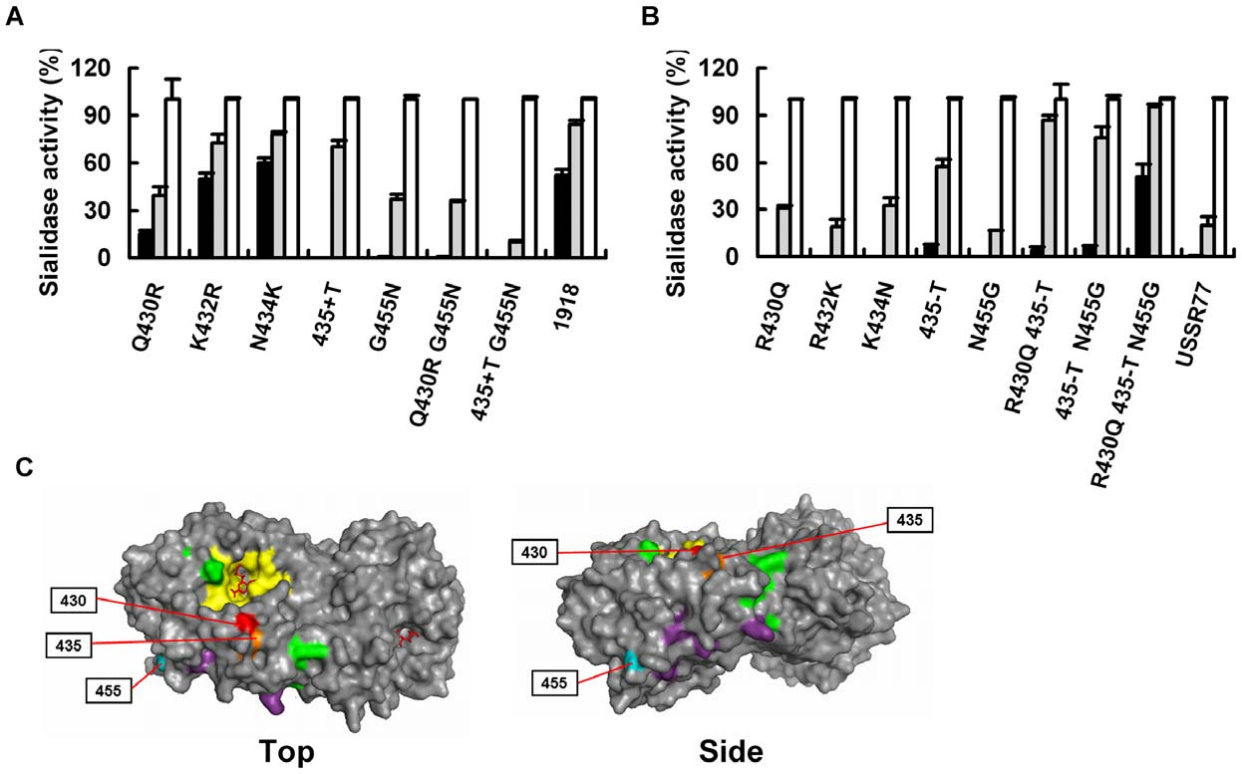
Determinant residues in the low-pH stabilities of sialidase activities of the 1918 and USSR77 NA. A–B, The low-pH stabilities of sialidase avtivities of mutated NAs from the 1918 virus (**A**) or USSR77 (B). “+T” and “-T” indicate insertion and deletion of Thr, respectively. C, Location of amino acid residues at positions 430 (red), 435 (orange) and 455 (light blue) on the NA dimer structure. Residues in the active site (yellow) with zanamivir (stick model), calcium ion binding site (green) and subunit interface (purple and light blue) are shown in the structure.

To specify determinant residues in the low-pH stability of the 1918 virus or recovered residues of low-pH stability in the low-pH-unstable USSR77NA, we generated 6 chimeric N1 NA genes between the 1918 virus and USSR77 (Figure 3A). Chimeras 2, 4 and 5 had low-pH stability like the 1918 virus, but other chimera NAs had no sialidase activity at pH 4.0 and 5.0 like USSR77NA (Figure 3B). Chimera 5 demonstrated that the NA amino acid region between 426 and 457 was essential for maintenance of the low-pH stability in the 1918 virus. Amino acid comparison within this region among 1918NA, USSR77NA, Tex91NA, Kaw176NA and Duck80NA suggested that each alteration at positions 430, 432, 434, and 455 (USSR77 N1 numbering) or deletion at position 435 in the 1918 virus NA could determine the low-pH stability (Figure 3C). Furthermore, we performed mutations between the 1918 NA and USSR77 NA at these positions. In the 1918 NA, insertion of Thr at 435 and alteration from Gly to Asn at 455 diminished sialidase activity at pH 4.0 (Figure 4A). Conversely, in the USSR77 NA, deletion of Thr at 435 and 2 alterations from Arg to Gln at 430 and from Asn to Gly at 455 maintained sialidase activity at pH 4.0 and 5.0 (Figure 4B). Single alteration at 430 or 455 in the 1918 NA also decreased the low-pH stability. However, alteration at 430 or 455 in USSR77 NA did not contribute to the low-pH stability. In the three-dimensional structure of A/Brevig Mission/1/18 NA, Gln and Thr at positions 430 and 435 were located near the active site, the calcium ion binding site and the subunit interface of native NA homotetramer. Gly at position 455 was one of the residues constituting the subunit interface (Figure 4C). Arg and Phe at positions 344 and 466 (N2 numbering), responsible for the low-pH stability of the pandemic A/Hong Kong/1/68 N2 NA, were also located near such a site [13]. Substitutions of these positions in N1 and N2 NA had a high potential to confer conformational change to the overall native NA structure, strongly suggesting that the low-pH stabilities of sialidase activity were dependent on the structure of the native NA.

### The low-pH-stable N1NA enhances virus replication

**Figure 5:**
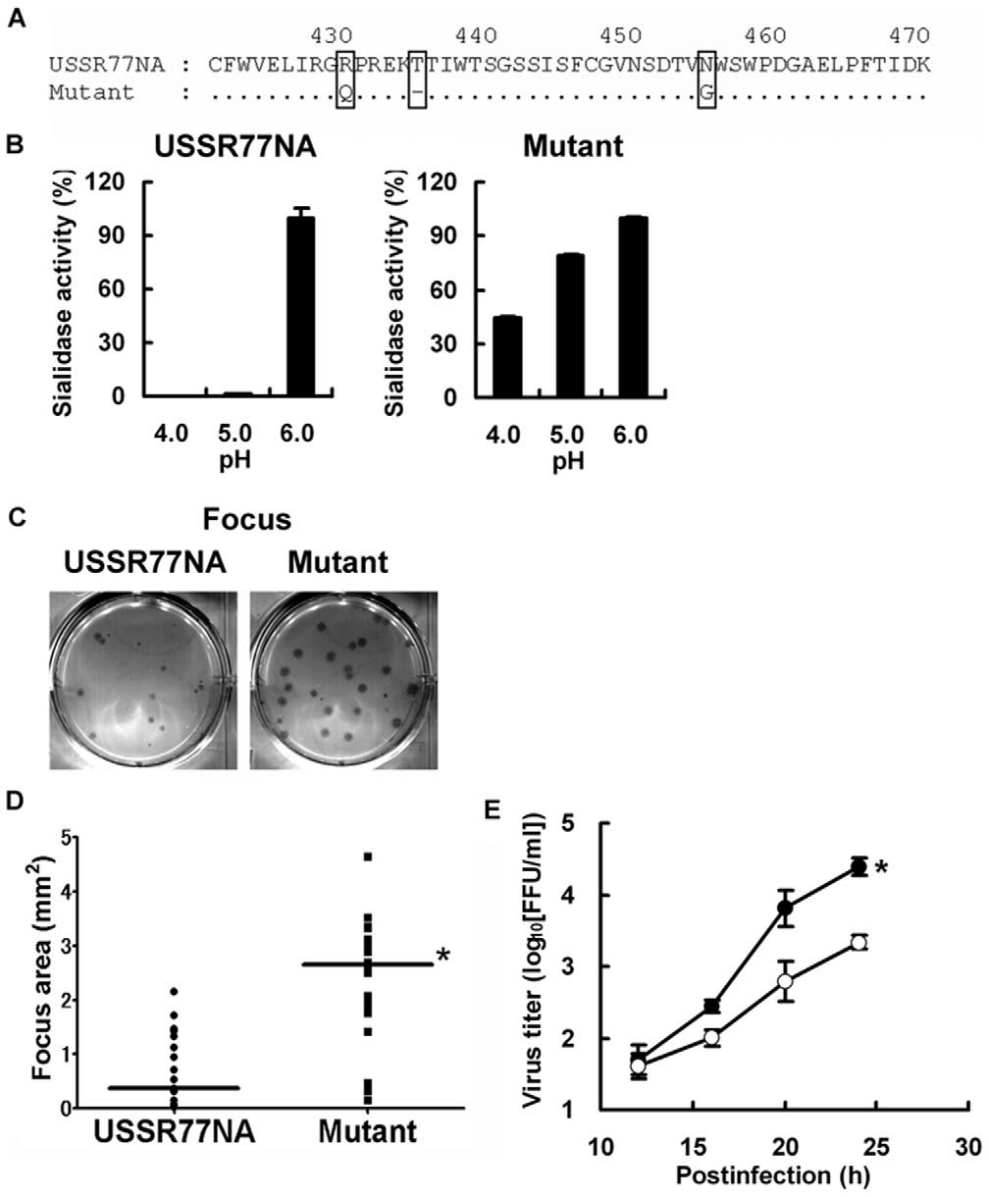
Efficient virus replication of the H1N1 virus possessing low-pH-stable NA. A, Mutated amino acid residues (USSR77 N1 numbering) in the low-pH-unstable NA (USSR77 NA) modified to the low-pH-stable NA (Mutant). “−” indicates deletion of an amino acid. B, Low-pH stabilities of sialidase avtivities of the viruses possessing USSR77NA or Mutant. C, Focus and plaque images of the viruses possessing USSR77NA or Mutant. D, Comparison of focus area between the viruses possessing USSR77NA (n = 28) and Mutant (n = 23). Bar indicates median. *, *p*<0.01 Mann-Whitney *U* test. E, Comparison of virus replication between the viruses possessing USSR77NA (closed circle, n = 3) and Mutant (open circle, n = 3). *, *p*<0.01 unpaired Student's *t* test.

We have found that low-pH stability of pandemic virus N2 NA increases virus replication [10]. To evaluate the contribution of low-pH-stable N1 NA to virus replication, we generated reverse genetics viruses (in the A/WSN/33 H1N1 background) possessing the low-pH-unstable wild-type USSR77 NA (USSR77NA) or the low-pH-stable N1 NA (mutant) by a deletion of Thr at 435 and 2 alterations from Arg to Gln at 430 and from Asn to Gly at 455 of the USSR77 NA (Figure 5A). As expected, the mutant had low-pH stability but USSR77NA did not (Figure 5B). Focus and plaque sizes of the mutant were larger than those of USSR77NA (Figure. 5C). Statistical analysis of focus area showed a significant difference between USSR77NA and the mutant (Figure 5D). Low-pH stability of the mutant significantly enhanced virus replication, coinciding with results for focus and plaque sizes (Figure 5E). These results demonstrate that the low-pH stability of N1 NA in the 1918 virus contributes to virus replication.

Characterization of the 1918 pandemic virus reconstructed by reverse genetics suggests that the 1918 NA activity facilitates HA cleavage [5]. The A/WSN/33 N1 NA abrogates the need for trypsin by conversion of plasminogen to plasmin, which facilitates HA cleavage, resulting in increased virus replication [18]. However, the NA-dependent spread of the 1918 virus was not due to plasminogen recruitment and NA facilitating HA cleavage by the 1918 NA [19]. Avian influenza viruses have both strong NA hemadsorption activity to chicken red blood cells and efficient hydrolysis of macromolecular substrates, but the NA of the 1918 virus differed from avian N1 NAs by reduced hemadsorption activity and less hydrolysis of macromolecular substrates. The NA of the 1918 virus might have already differed from its putative avian ancestor in birds [20]. This speculation is supported by the intermediate degree of low-pH stability in the 1918 NA between the human NA and the avian NA (Figure 2). Our study is the first research showing that the nature of the 1918 NA correlates to enhanced virus replication.

The low-pH stability of the 1918 NA supports the origin from avian NA. Determinants (deletion of Thr at position 435 and Gly at position 455) of the low-pH stability of the 1918 virus N1 NA were also conserved in highly pathogenic H5N1 viruses [21]. Further studies are needed to confirm that H5N1 virus NAs are predicted to have low-pH stability which contributes to the viral replication efficiency and a pandemic potential.

## Materials and Methods

### Viral NA genes and plasmids

The NA gene of the 1918 virus inserted into the pHH21 plasmid vector and the NA gene of the A/WSN/33 virus (WSN) inserted into the pCAGGS/MCS expression plasmid vector were prepared as described previously [10], [13]. RNA genomes of influenza A viruses, A/Texas/91 (H1N1), A/USSR/92/77 (H1N1), A/Kawasaki/176/02 (H1N1) and A/duck/849/3/80 (H4N1), were isolated and converted to cDNAs. Each NA gene was inserted into the multicloning site between the *Eco*RI site and the *Xho*I site of the pCAGGS/MCS expression plasmid vector.

### Measurement of sialidase activity on cell-expressed NA

Human embryonic kidney 293T cells [22] were maintained in high-glucose Dulbecco's modified medium supplemented with 10% fetal bovine serum (FBS). 293T cells at 70% confluency in a 24-well tissue culture plate were transfected with 1 µg/well of each pCAGGS expression vector using TransIT-293 (Mirus, Madison, WI). After incubation for 24 h at 37°C, the transfected cells were collected by suspension with phosphate-buffered saline (PBS; 1.2 ml/well). Fifty microliters of each cell suspension was transferred into new microtubes and centrifuged. The cell pellets were incubated with 50 µl of 10 mM acetate buffer (pH 4.0, 5.0, and 6.0) at 37°C for 10 min and then reacted by addition of 2.5 µl of 2 mM 2′-(4-methyl-umbelliferyl)-*N*-acetylneuraminic acid (4MU-Neu5Ac; Sigma-Aldrich Corp., Missouri) at 37°C for 30 min. The reaction was stopped by addition of 200 µl of 100 mM sodium carbonate buffer (pH 10.7). Then fluorescent intensity (Ex, 355 nm; Em, 460 nm) was measured with a Wallac 1420 ARVOsx multi-label counter (Perkin Elmer, Waltham, MA). Sialidase activities (%) of the cell-expressed NA were expressed as a percentage of that at pH 6.0 of viral NA.

### Flowcytometric analysis

The transfected cells in a 24-well plate were collected by suspension with PBS (500 µl/well). After fixation with 4% paraformaldehyde at room temperature for 10 min, NA expressed on the cell surface was detected by rabbit anti-N1 NA polyclonal antibody (Abcam, Cambridge, UK; ab21304) and fluorescein isothiocyanate (FITC)-conjugated goat anti-rabbit IgG antibody (Invitrogen, Carlsbad, CA). The mean fluorescent intensity (MFI) of the cells was measured using FACS Canto II flow cytometer (BD, Franklin Lakes, NJ). Relative MFI (%) of NA-expressing cells were expressed as a percentage of that of mock-treated cells.

### Generation of chimeric and mutated NAs

Utilizing shared restriction enzyme sites for *Pst*I, *Vsp*I, and *Bal*I (at the 1918 virus N1 NA nucleotide positions 974, 1295, and 1390) among the pCAGGS plasmid vectors containing the low-pH stable 1918 virus NA and the low-pH unstable USSR77 NA, we generated 6 chimeric constructs (Figure 3A).

NA genes were mutated according to the procedure of the QuickChange II Site-directed Mutagenesis kit (Stratagene, La Jolla, CA) by using respective mutated primer pairs. The mutated NA genes generated on the pGEM-T easy vector (Promega, Madison, WI) or pHH21 were amplified and inserted into pCAGGS/MCS multi cloning sites between *Eco*RI and *Xho*I.

### Generation of reverse genetics viruses

Reverse genetics in the A/WSN/33 H1N1 background [23] was performed using the pHH21 vector containing the wild-type USSR77 NA gene or mutated USSR77 NA gene (a deletion of Thr at 435 and 2 alterations from Arg to Gln at 430 and from Asn to Gly at 455), instead of the pHH21 vector containing the WSN NA gene. Viruses were propagated using Madin-Darby canine kidney (MDCK) cells [24] in a serum free medium (SFM), Hybridoma-SFM (Invitrogen Corp., Carlsbad, CA) containing acetylated trypsin (2.0 µg/ml).

Concentrated viruses were diluted with 100 mM acetate buffer (pH 4.0 and 5.0) or 100 mM phosphate buffer (pH 6.0). Viruses (protein of 300 ng) were incubated in each buffer at 37°C for 10 min and then reacted by addition of 5.0 µl of 2 mM 4MU-Neu5Ac at 37°C for 30 min. The reaction was stopped by addition of 100 µl of 100 mM sodium carbonate buffer (pH 10.7). Then fluorescent intensity was measured. Sialidase activities (%) were expressed as a percentage of that at pH 6.0 of viral NA.

### Focus assay and virus titration

MDCK cells were maintained in Eagle's minimum essential medium supplemented with 5% FBS. Confluent monolayer MDCK cells on a 6-well tissue culture plate were incubated with log dilutions of the virus in SFM for 1.0 h at 37°C. The infected monolayers were then overlaid with a solution of SFM containing acetylated trypsin (2.0 µg/ml) and 0.8% agarose. The monolayers were incubated at 37°C for 72 h. Cells were fixed with 2.0 ml/well of ethanol: acetic acid (v/v = 5 ∶ 1) solution at 4°C overnight. Viral antigens in cells were reacted with anti-influenza A virus nucleoprotein monoclonal antibody (4E6) for 30 min and then with horseradish peroxidase-conjugated goat anti-mouse IgG+M (Jackson Immuno Research, West Grove, PA) for 30 min at room temperature. Viral foci were stained as previously described [25]. Area of stained focus was measured using Image J release 1.40 g (National Institutes of Health, USA, http://rsb.info.nih.gov/ij/).

### Virus replication

Confluent monolayer MDCK cells on a 12-well tissue culture plate were infected with 0.0001 of multiplicity of infection (MOI) of viruses at 37°C for 1.0 h. After washing viruses with 500 µl of PBS, cells were incubated in SFM containing acetylated trypsin (2.0 µg/ml) at 37°C. At 12, 16, 20 and 24 h, supernatants of cells were recovered. Virus titers in the supernatants were measured by focus assay.

### Three-dimensional structure

Structure of the NA dimer (PDB ID, 3B7E) was depicted using DeLano Scientific PyMOL release 1.11 (DeLano,W.L. The PyMOL Molecular Graphics System, http://pymol.sourceforge.net).

## Supporting Information

Figure S1 **Effect of Ca^2+^ on Low-pH stability of sialidase activity of the 1918 virus NA.** 293T cells genetically expressing each N1 NA from USSR77 and 1918 viruses were incubated with 10 mM acetate buffer (pH 4.0, 5.0, and 6.0) containing 5 μM CaCl_2_ at 37°C for 30 min and sialidase activities were measured. Sialidase activities were expressed as a percentage of each activity at pH 6.0. As a control, 10 mM acetate buffer (pH 4.0, 5.0, and 6.0) without CaCl_2_ was used.
(TIF)
