# Supplementary figures and images for: The Low-pH Stability Discovered in Neuraminidase of 1918 Pandemic Influenza A Virus Enhances Virus Replication
Source: PLoS One. 2010 Dec 9;5(12):e15556. doi: 10.1371/journal.pone.0015556 (PMC3000343; doi:10.1371/journal.pone.0015556)

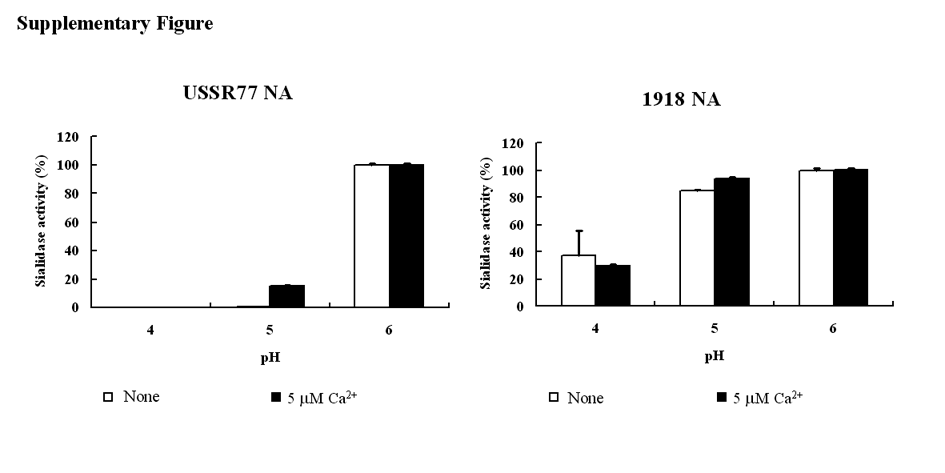

Supplement: Figure S1 — Effect of Ca2+ on Low-pH stability of sialidase activity of the 1918 virus NA. 293T cells genetically expressing each N1 NA from USSR77 and 1918 viruses were incubated with 10 mM acetate buffer (pH 4.0, 5.0, and 6.0) containing 5 μM CaCl2 at 37°C for 30 min and sialidase activities were measured. Sialidase activities were expressed as a percentage of each activity at pH 6.0. As a control, 10 mM acetate buffer (pH 4.0, 5.0, and 6.0) without CaCl2 was used. (TIF) [file pone.0015556.s001.tif]
